# Chlorhexidine versus organoselenium for inhibition of *S. mutans* biofilm, an in vitro study

**DOI:** 10.1186/s12903-022-02049-w

**Published:** 2022-01-20

**Authors:** Abdul Seguya, Mohamed Mowafy, Ahmed Gaballah, Abbas Zaher

**Affiliations:** 1 Department of Orthodontics, Faculty of Dentistry, Alexandria University, Champollion St., Azarita, P. O. Box: 21521, Alexandria, Egypt; 2 Department of Microbiology, Medical Research Institute, Alexandria University, Alexandria, Egypt

**Keywords:** Organoselenium, Selenium, Chlorhexidine diacetate, Chlorhexidine varnish biofilm formation, White spot lesions, *S. mutans*

## Abstract

**Background:**

Chemical Plaque control by antimicrobial agent application can defend the teeth against caries. *S. mutans* is considered the main etiologic factor for caries. This was an in vitro study to compare between the efficacy of chlorhexidine diaceteate varnish, and an organoselenium sealant, to prevent *S. mutans* biofilm formation on human teeth.

**Methods:**

Fourty five premolars extracted for orthodontic purposes were randomly divided into 3 groups of 15 teeth each. One control group and two test groups, chlorhexidine diaceteate varnish and an organoselenium sealant. The teeth were autoclaved before *S. mutans* biofilm was induced on to each in their respective groups. The reading T1 was taken for each tooth to assess the number of *S. mutans* attached in order to compare for differences in surface area among the 3 groups. The respective test materials were applied onto the teeth and biofilm induced onto them in their respective groups. The reading T2 was taken for the 2 test groups. The 3 groups were then subjected to aging for a period equivalent to 5 months before the biofilm was induced to take the reading T3 for the number of *S. mutans*. We used vortexing of the teeth to disrupt the biofilm at time points T1, T2 and T3. *S. mutans* count was then done using PCR.

**Results:**

There were significantly lower *S. mutans* counts in the control group as compared to the chlorhexidine diacetate group at T3.There were no other statistically significant differences found.

**Conclusion:**

Both organoselenium and Chlorhexidine diacetate do not inhibit *S. mutans* biofilm attachment onto the teeth.

## Introduction

The oral cavity has about 700 known species of bacteria, including 140 dominating ones that make the multi-species biofilm, dental plaque [1]. Biofilms are clusters of microbes which are embedded in organic matrixes of self-made patterns of extracellular polysaccharides, proteins and DNA. The biofilm shields bacteria from dehydration, host defences, predators and donates boosted resistance to antimicrobial materials. Further, the polysaccharides provide a constant nutrient supply for the bacteria to produce acids in times of starvation [2]. *Streptococcus mutans* plays a special role in the development of the dental plaque’s biofilm and caries etiology, mainly due to the interaction with other streptococci [3], and the ability to produce glucosyltransferase (GTF)—an enzyme that participates in the synthesis of glucans that make the colonization of dental surfaces easier [4]. According to the specific Plaque hypothesis, *S. mutans* is predominantly responsible for dental decay [5].

Chemical plaque control, by antimicrobial agents application, can defend the teeth against negative effects, to reduce the accumulation of plaque, and to better oral health [6–8].

White Spot Lesions (WSLs) are a significant clinical problem in relation to treatment with fixed orthodontic appliances [9]. The overall prevalence of WSL among orthodontic patients has been reported to be between 26 and 89% [10]. WSLs can be seen within 4 weeks after fixed orthodontic appliances treatment commencement [11], if preventive measures are not established [12].

Orthodontic appliances make cleaning difficult hence the accumulation of bacterial biofilms on dental surfaces [13], whose bacteria produce acids from the fermentation of food debris leading to the carious process by means of demineralization of hydroxyapatite crystals of dental enamel.

Methods to prevent or minimize WSL development during orthodontic treatment can be divided into those for which patient compliance is required and those for which patient compliance is not required. Compliance based methods include tooth brushing, flossing and the compliance independent methods include applying of cavity varnishes, sealants among others. Compliance-independent methods seem to be more suitable for minimizing WSLs [14]. Among the intraorally used antimicrobial agents, chlorhexidine (CHX) has been one of the most frequently used antiplaque chemical products against oral bacteria [15]. There can be different compliance problems with the CHX-containing mouth rinse which can be avoided by use of the varnish [15]. Ivoclar Vivadent came up with a varnish called cervitec plus. An organo-selenium compound DenteShield™ (SeleneBio, Austin Texas) was introduced on the dental market. This compound covalently adheres to different biomaterials to inhibit bacterial biofilm [16]. There have been studies investigating *Streptococcus mutans* biofilm formation on organoselenium discs but non that used actual teeth in their study model. Also none applied thermal and mechanical aging. There have been in vivo studies investigating plaque formation around Organoselenium sealants but these were using a multiorganism vehicle. Also the studies used only phenotypic methods i.e., culture using standard plate count to assess bacterial count. A method which has been shown to not be as sensitive as molecular methods like PCR. Further still, there have been several studies investigating Chlorhexidine varnishes but non that compared between their efficacies with organoselenium sealants.

Therefore, the aim of this work is to compare the efficacy of a chlorhexidine containing varnish (Cervitec Plus), and a selenium containing sealant (DenteShield™) in the inhibition of biofilm formation on tooth surfaces immediately after application of the materials and 5 months after aging factor.

## Methods

It is a randomized controlled in-vitro study to compare between the efficacy of the application of a chlorhexidine diacetate varnish (cervitec plus) and that of a selenium containing sealant (DenteShield™)in the prevention of biofilm formation on teeth.

The study was conducted following the approval of the Institutional review board of the Faculty of Dentistry, Alexandria University. (IRB: 00010556-IORG: 0008839). All methods were carried out in accordance with the CRIS guidelines and Regulations.

The study was conducted at the Orthodontic and Biomaterials Departments at the Faculty of Dentistry, Alexandria University as well as the Microbiology department of the Medical Research Institute, Alexandria University.

### Sample preparation

The sample size estimate was 30 premolars with at least 10 premolars per group. This was calculated using GPower software version 3.1.9.2 using an error of 0.05 and a study power of 80% [17]. To cater for any damage or loss during the study, 45 premolars that had been extracted for orthodontic purposes were used and divided equally into three groups using simple randomization. Informed consent was sought from each individual or their guardians before taking their teeth. Only Premolars with an intact enamel surface without caries, restorations or cracks were included in the study.

Immediately after extraction, the teeth were thoroughly cleaned under running water. All calculus and soft tissue remnants were removed using a hand scaler. The teeth were then stored in distilled water at room temperature to avoid dehydration.

Using simple randomization, the teeth were divided into three groups I, II, III of 15 teeth each: I—Control. II—Chlorohexidine varnish. III—Oganoselenium sealant.

**Fig. 1:**
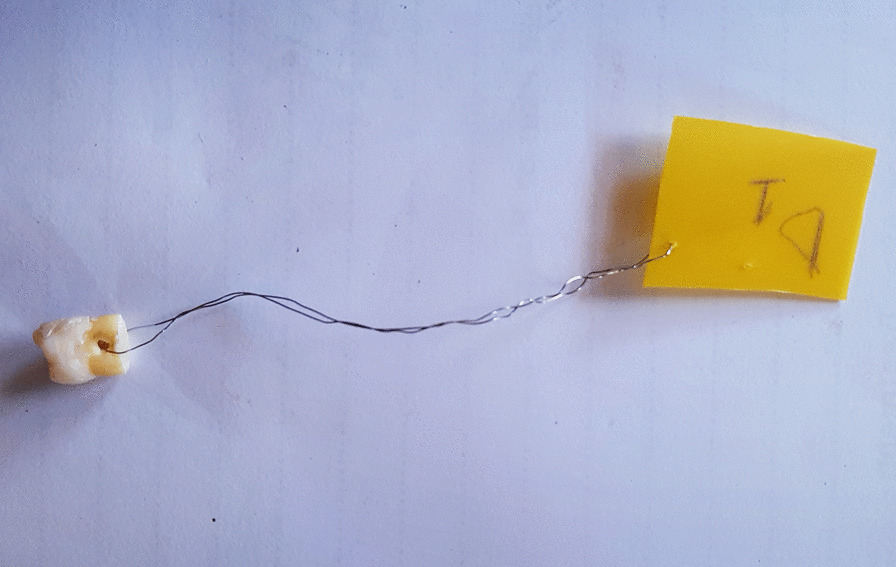
Sample tooth as was used in the experiment

The apical third of the root was sectioned off. A hole was drilled near the apical part by means of a rose-head bur on a high speed hand piece. A ligature wire was passed through this hole to allow handling of the teeth during the experimental period and to attach tags that carried unique numbers to the teeth. The apical part of the tooth was sealed with composite as shown in Fig. 1.

**Fig. 2:**
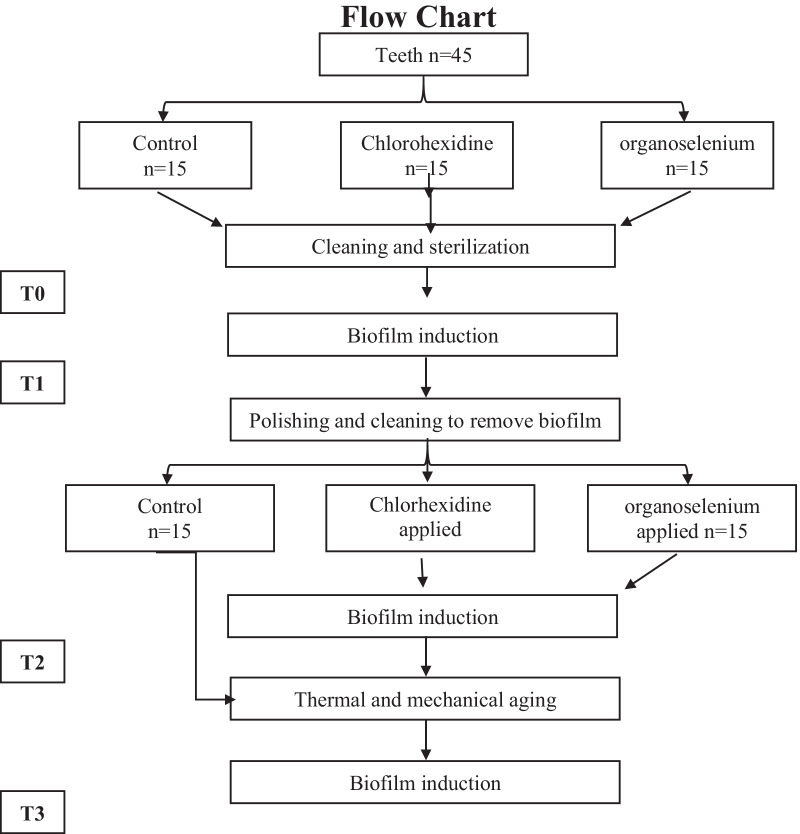
Flow chart of how the experiment proceeded

At the onset of the study, the enamel surface was cleaned and polished with a rubber prophylaxis cup at low speed using fluoride-free pumice and water. The teeth were then thoroughly rinsed, Placed in a sterile bag and autoclaved. Each tooth was assigned a number from 1 to 15 for identification purposes and to facilitate re-examination in their respective groups. The experiment proceeded as shown in the flow chart Fig. 2.

### Isolation of *Streptococcus mutans*

**Fig. 3:**
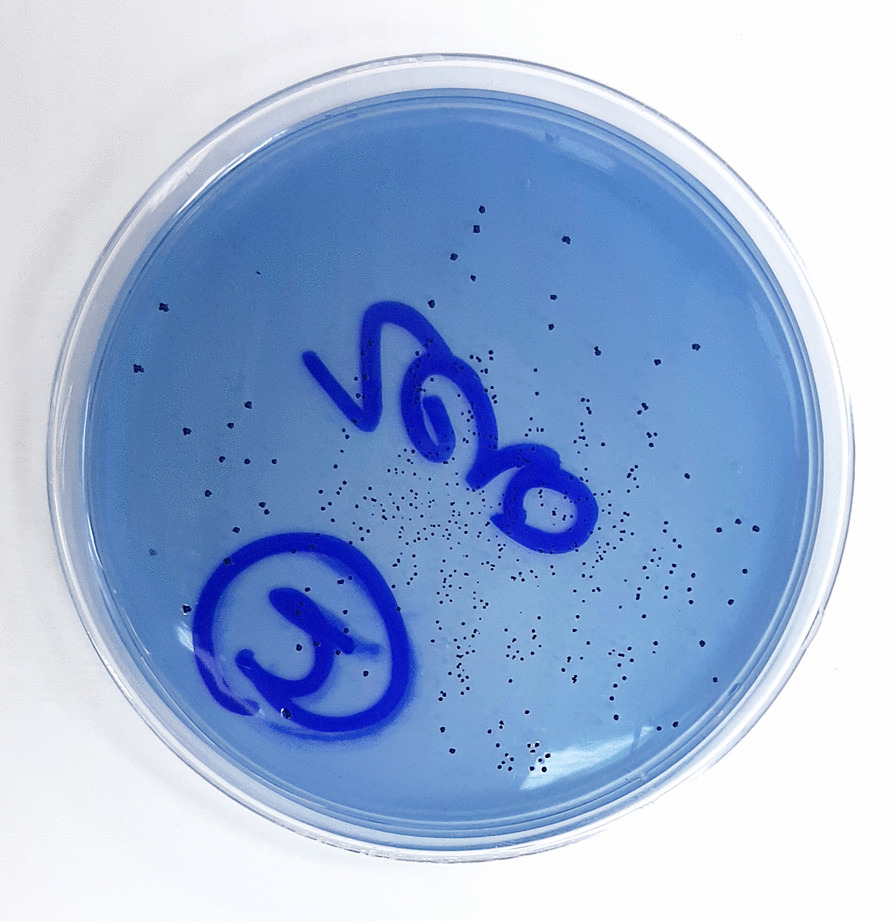
A plate of isolated *S. mutans* on MSB agar

**Fig. 4:**
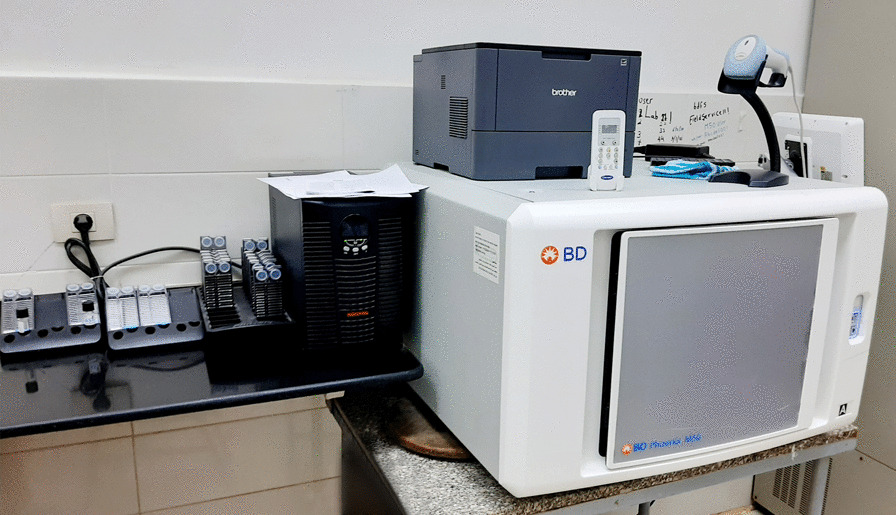
BD Phoenix™ automated identification and susceptibility testing system

A sample from dental plaque was collected on a tip of sterile wooden toothpick from myself. Plaque was dispersed in 1 mL of sterile phosphate buffer saline and vortexed to obtain a homogenous suspension. The suspension was then plated on modified MSB agar (Himedia, India) Fig. 3 and incubated anaerobically at 37 °C for 48 h. BD Phoenix™ automated identification and susceptibility testing system (Fig. 4) was used for identification of the colonies. The identified *S. mutans* Isolate was stored at − 80 °C in LB-glycerol culture till needed [18]. It was to be used for initiating biofilm formation during the experiments and to prepare the standard curve for real-time PCR.

### Standard suspensions of each isolate containing 10^7^***S. mutans***/mL were prepared

**Fig. 5:**
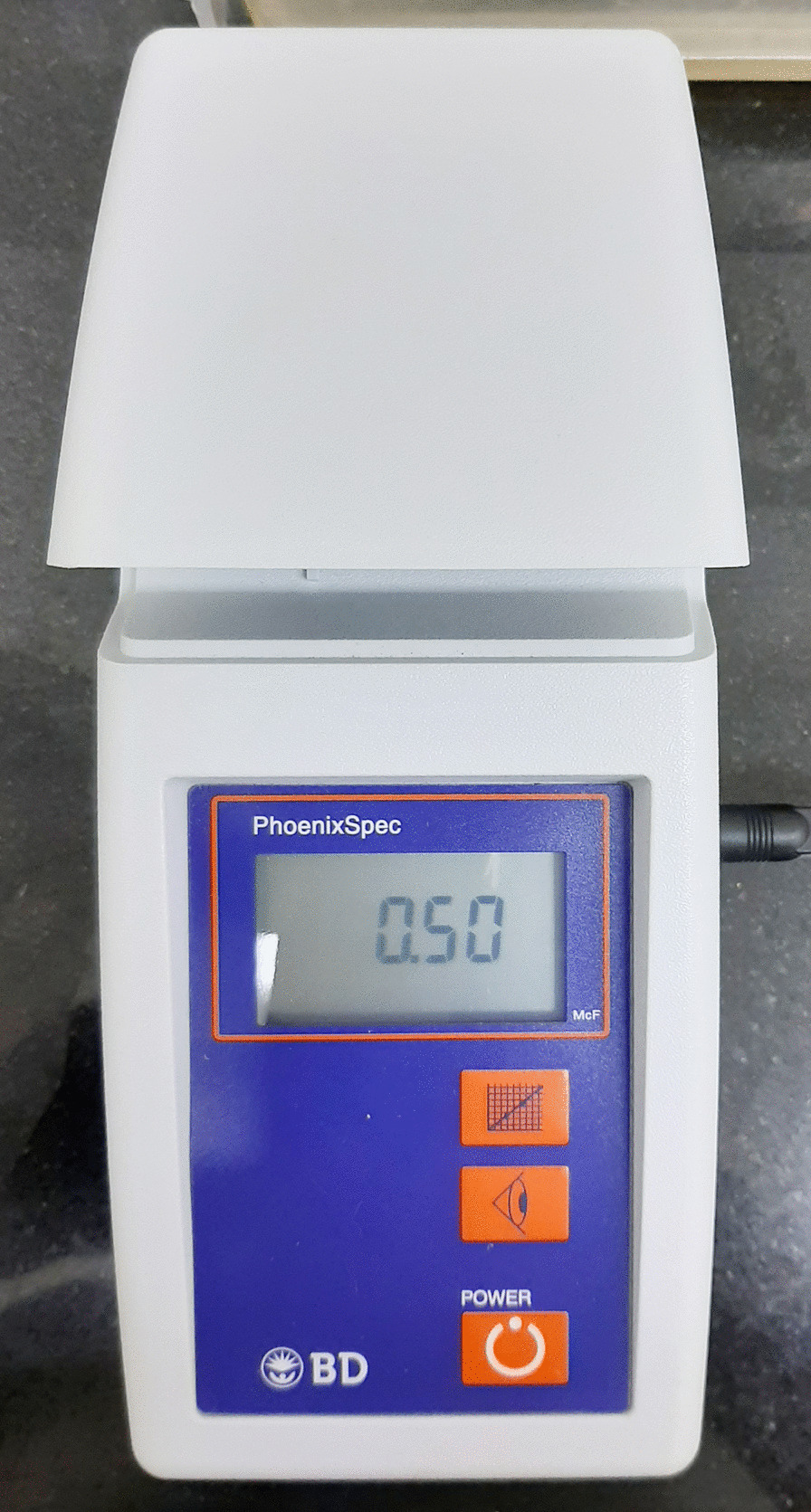
The BD PhoenixSpec™ nephelometer for reading the mcfarland standard

A 10 μL aliquot of each thawed and homogenized sample, that was stored previously, was streaked with a loop onto Petri dishes containing the MSB (Himedia, India) agar. The aliquots were seeded onto brain heart infusion (BHI) agar and incubated for 48 h at 37 °C in a candle jar. After incubation, the microorganisms were cultured on blood agar (Oxoid, USA) for at least 18 h at 37 °C in a candle jar. To confirm the identity of *S. mutans*, BD Phoenix™ automated identification and susceptibility testing system was used. The bacterial cultures were prepared using Mueller–Hinton broth (Oxoid, USA). Briefly 5 mL of sterile Mueller–Hinton broth inoculated with colonies of *S. mutans* to a concentration of 0.5McFarland standard as read by the BD PhoenixSpec™ nephelometer (Fig. 5) to make the standard *S. mutans* suspension [19]. This concentration was later diluted 1 to 10 to get 10^7^ cells/mL.

### Induction of biofilm formation by *S. mutans*

The biofilms were grown on specimens of human teeth. The specimens were placed in sterile glass test tubes, suspended in each test tube by means of a ligature wire, with 1.8 mL sterile broth and were inoculated with 0.2 mL *S. mutans* standard suspensions. The ligature supports were made to suspend the specimens, with the crowns submerged to the cementoenamel junction, to simulate the oral cavity. They were then placed in candle jar and left in the incubator at 37 °C for 24 h.

**Fig. 6:**
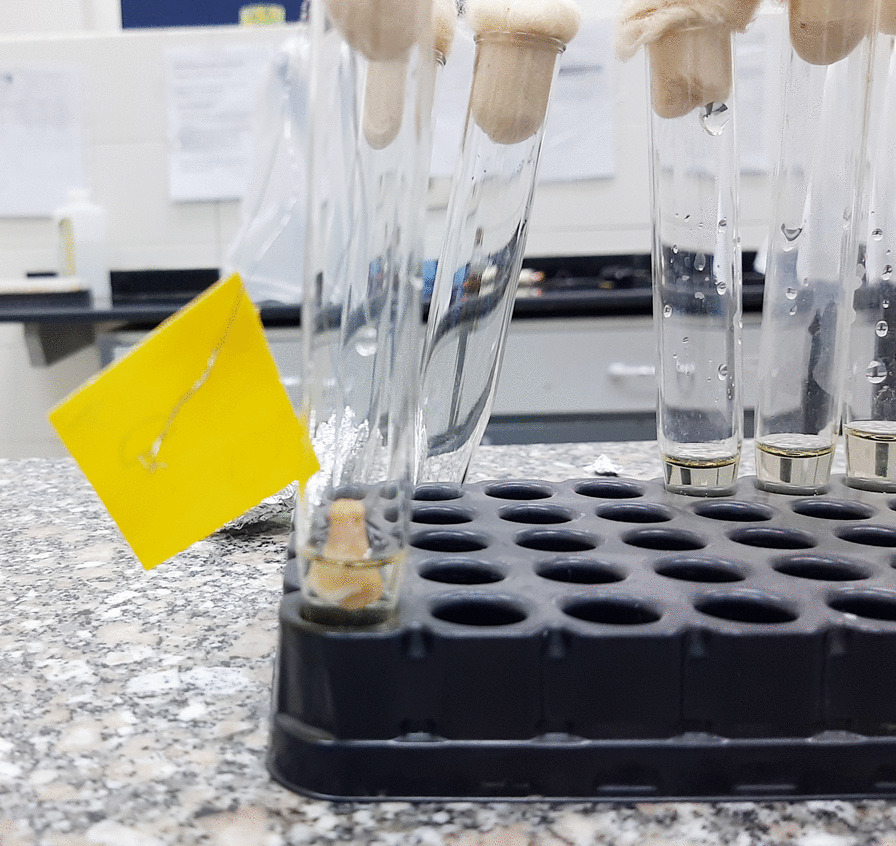
Tooth in test tube as was used in the experiment

To induce biofilm formation each tooth was placed alone in a glass test tube with 2 mL Mueller Hinton broth inoculated with 10^7^ *S. mutans* cells/mL as in Fig. 6. Test tubes were incubated in a candle jar at 37 °C for 24 h.

### Thermal and mechanical aging

Teeth were brushed for 3000 strokes using brushing machine with soft bristled brushes with a constant force applied to each brush simulating the oral cavity for 5 months [20].

Teeth were further subjected to a thermocycling protocol (SD Mechatronik, Feldkirchen-Westerham, Germany) and were cycled in water between 10 and 50 °C with a dwell time of 30 s and a transfer time of 10secs for 500 cycles [21].

### Assessment of the number of *S. mutans* attached on to the surface of the teeth

Real time PCR were used. The investigator carrying out the PCR reactions was blinded to which samples belonged to which group. At each stage, the specimens were washed by dipping in physiologic saline, placed in tubes with 10 mL sterile physiologic solution (0.85% NaCl) and vortexed for 3 min.

### Extraction of chromosomal DNA

The extraction of DNA was as described by Oho et al. [22]. Briefly 5 mL of bacterial suspension in saline were centrifuged at 13,000 rpm for 1 min to obtain bacterial pellet. Pellet was resuspended in 200 µL of Tris–EDTA buffer and incubated in boiling water bath for 10 min. After centrifugation at 13000 rpm for 10 min, the supernatant was used as a source for bacterial DNA.

### Real time PCR

**Fig. 7:**
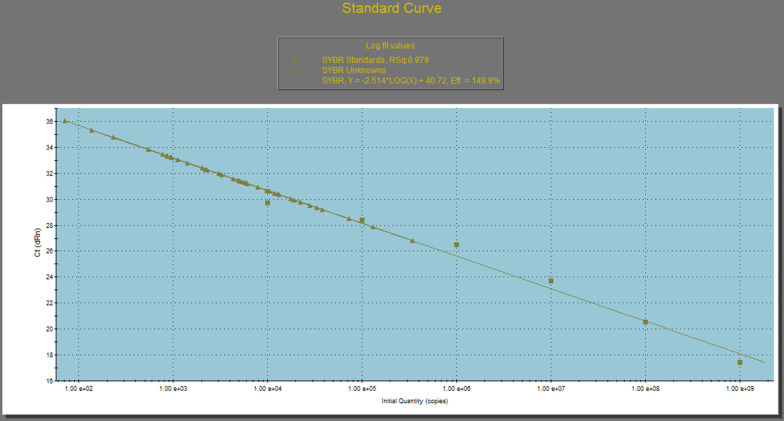
The standard curve as was generated in our experiment

The PCR Technique was as described by Yano et al. [23]. PCR was used to quantify *S. mutans* in biofilm molecularly. Primer Pairs were selected targeting a conserved region of *S. mutans*. The sequence of the forward gtfB primer, Smut3368-F, was: 5′-GCCTACAGCTCAGAGATGCTATTCT-3′. The sequence of the reverse gtfB primer, Smut3481-R, was: 5′- GCCATACACCACTCATGAATTGA-3′. The PCR was performed in 15 µL final reaction volume containing 7.5 µL sybr green master mix, (Thermoscientific, Lithuana), 0.5 µL of each primer (10 pmol/µL) and 1 µL of DNA extract, 5.5 µL of distilled water with 0.07 Rox dye. A standard curve, Fig. 7, was instituted using a series of tenfold serial dilutions liquid culture. PCR was conducted using Strategene mx 3000P a real time PCR detection system with the following conditions: 95 °C for 15 min for enzyme activation followed by 40 cycles of two temperature PCR with denaturation at 95 °C for 15 s followed by annealing and extension at 60 °C for 40 s.

## Results allocation

Quantification of *Streptococcus mutans* attached to the surface of the teeth was determined at different time points as follows:

- **T0:** This point was after cleaning and autoclaving of the teeth. It is to confirm the sterility before starting the experiment. It was assumed to be 0 for each tooth based on the indicator color change on the sterilization bags.
- **T1:** After inducing biofilm formation on all the teeth groups included in this study. At this point the ability of the isolated organism to form a biofilm of the groups was assessed, this also allowed for statistical comparison for differences between the 3 groups.
- **T2:** On the samples from T2 above, biofilm formation was induced on the respective groups. At this point the ability of the materials to inhibit biofilm formation was assessed.
- **T3:** The samples from T3 were then subjected to mechanical and thermocycling as described previously. Biofilm formation was induced before the reading T3 was taken. At this point the durability of the materials to inhibit biofilm formation was assessed.

### Statistical analysis of the data

Data were fed to the computer and analyzed using IBM SPSS software package version 20.0. (Armonk, NY: IBM Corp). The Kolmogorov–Smirnov test was used to verify the normality of distribution Quantitative data were described using range (minimum and maximum), mean, standard deviation, median and interquartile range (IQR). Significance of the obtained results was judged at the 5% level. The tests used were: 1—Kruskal Wallis test 2—Friedman test.

### Results from PCR analysis

There were no statistically significant differences between the three groups before application of any of the test materials onto the teeth in their respective groups at T1.

There was also no statistically significant difference between the organoselenium sealant and chlorhexidine diacetate varnish groups just after application of the test materials to their respective groups at T2 nor after thermal and mechanical aging at T3.

However, after application of the materials followed by thermal and mechanical aging at T3, the control group showed a significantly lower mean number of *Streptococcus mutans* counts of 7.29 × 10^2^ as compared to chlorhexidine diacetate varnish group mean count of 6.94 × 10^3^.There were no other significant differences (Table 1).

**Table 1:** Comparison between the three studied groups according to PCR

PCR	Control	Chlorhexidine varnish	Organoselenium sealant	Test of Sig.	*p*
T1	(n = 13)	(n = 14)	(n = 15)	H = 4.878	0.087
Min.–Max.	1.010E+02–3.776E+04	7.193E+01–2.219E+04	2.344E+02–7.318E+04		
Mean ± SD.	6.458E+03 ± 1.294E+04	6.115E+03 ± 6.998E+03	1.070E+04 ± 1.837E+04		
Median	9.601E+02	3.655E+03	4.318E+03		
IQR	8.656E+02–2.275E+03	3.676E+02–9.902E+03	3.303E+03–8.043E+03		
T3	(n = 13)	(n = 15)	(n = 14)	H = 4.135	0.127
Min.–Max.	1.010E+02–3.776E+04	4.232E+01–1.923E+04	5.351E+02–3.441E+05		
Mean ± SD.	6.458E+03 ± 1.294E+04	4.527E+03 ± 5.818E+03	3.796E+04 ± 9.442E+04		
Median	9.601E+02	1.428E+03	4.843E+03		
IQR	8.656E+02–2.275E+03	6.091E+02–7.853E+03	2.095E+03–1.051E+04		
T4	(n = 15)	(n = 15)	(n = 14)	H = 6.560^*^	0.038^*^
Min.–Max.	9.650E+01–1.697E+03	3.722E+01–2.462E+04	1.048E+02–1.194E+04		
Mean ± SD.	7.290E+02 ± 5.245E+02	6.941E+03 ± 8.358E+03	2.670E+03 ± 3.084E+03		
Median	5.450E+02	2.707E+03	1.981E+03		
IQR	3.544E+02–1.007E+03	4.701E+02–1.315E+04	4.256E+02–3.135E+03		
Sig. bet. grps.	*p*_1_ = 0.016^*^, *p*_2_ = 0.052, *p*_3_ = 0.670				

*IQR* inter quartile range, *SD* standard deviation, *H* H for Kruskal Wallis test, Pairwise comparison bet. Each 2 groups was done using Post Hoc Test (Dunn's for multiple comparisons test)

*p*: *p* value for comparing between the three studied groups

p_1_: *p* value for comparing between Control and Chlorohexidine varnish

*p*_2_: *p* value for comparing between Control and Organoselenium

*p*_3_: *p* value for comparing between Chlorohexidine varnish and Orhanoselenium

*Statistically significant at *p* ≤ 0.05

There was no statistically significant difference with in the Chlorhexidine diacetate (Cervitec Plus) varnish group nor with in the organoselenium (Denteshield™) group at T1, T2 and T3 (Table 2).

**Table 2:** Comparison between the three studied readings according to PCR in each group

PCR	T1	T3	T4	Fr	*p*
Chlorohexidine varnish	(n = 14)	(n = 15)	(n = 15)	1.000	0.607
Min.–Max.	7.193E+01–2.219E+04	4.232E+01–1.923E+04	3.722E+01–2.462E+04		
Mean ± SD.	6.115E+03 ± 6.998E+03	4.527E+03 ± 5.818E+03	6.941E+03 ± 8.358E+03		
Median	3.655E+03	1.428E+03	2.707E+03		
IQR	3.676E+02–9.902E+03	6.091E+02–7.853E+03	4.701E+02–1.315E+04		
Organoselenium sealant	(n = 15)	(n = 14)	(n = 14)	5.692	0.058
Min.–Max.	2.344E+02–7.318E+04	5.351E+02–3.441E+05	1.048E+02–1.194E+04		
Mean ± SD.	1.070E+04 ± 1.837E+04	3.796E+04 ± 9.442E+04	2.670E+03 ± 3.084E+03		
Median	4.318E+03	4.843E+03	1.981E+03		
IQR	3.303E+03–8.043E+03	2.095E+03–1.051E+04	4.256E+02–3.135E+03		

*IQR* inter quartile range, *SD* standard deviation, *Fr* Friedman test

*p*: *p* value for comparing between the three studied readings

## Discussion

White spot lesions or enamel demineralization are an undesirable common side effect of fixed orthodontic appliances. Many of these lesions are irreversible thus prevention of these lesions is more prudent than managing them [24]. Also dental caries is a progressive and recurrent disease [2]. Treatment of demineralized WSLs is another option after debonding but it is riddled with lack of reliable evidence as regards their effectiveness over a long time [15]. *Streptococcus mutans* is considered to be the primary etiologic factor of plaque [25]. Chemical plaque control methods that don’t require patient compliance are an exciting notion in the fight against white spot Lesions. This was an in vitro study to test the ability of both a chlorhexidine diacetate varnish, and an organoselenium sealant, to inhibit *Streptococcus mutans* biofilm formation on teeth using PCR for the bacterial count. It went ahead to compare the activity of the two materials before as well as after thermal and mechanical aging for a period equivalent to 5 months.

There were no statistically significant differences between the three groups before application of the test materials at T1 which implies that there are no differences in relative surface area.

Using the reading of the control group at T1 compared with the two test groups at T2, we found no statistically significant differences.

There were also no statistically significant differences between the chlorhexidine varnish group and the organoselenium group immediately after application of the test materials At T2 nor after thermal and mechanical aging at T3. This implies that both materials are equally as effective. Both materials are known to be bacteriostatic as well as bactericidal.

After thermal and mechanical aging at T3, the Organoselenium group had a mean bacterial count of 2.67 × 10^3^, which is not in agreement with Amaechi et al. [26] and Tran et al. [16] who found that no bacteria could attach to the surface of organoselenium sealants with 0.25% organoselenium in vitro. This could be explained by a number of hypotheses: Fitnak et al. [27] found the minimum inhibitory concentration of organoselenium (selol) for *Streptococcus mutans* to be 1.25% while Denteshield™ contains only 0.5% [26]. This could also be because the material was over cured as compared to the 20 s recommended by the manufacturer which could have further strengthened the bond between the selenium and resin polymer allowing for almost no leaching out of selenium. Also previous studies used plate count while this study made use of the PCR which assesses the total number of bacterial DNA present irrespective of vitality status. The previous in vitro studies also used discs which could have allowed for higher volumes of selenium leaching out as compared to the thin layer of sealant applied to the tooth surface. Also the stability of Denteshield™ has not been reported at temperatures of up to 50 °C. Amaechi et al. [26] found no plaque growth in the pits and fissures of his study group at 9 months. This could be because he used an in vivo study model and the method used to assess the presence of plaque was quantitative light induced fluorescence (QLF). Amaechi et al. [26] also did not report if he excluded patients with stained teeth. QLF is a method whose sensitivity is affected by the presence of certain bacteria like *P. ginvivalis*, *P. intermedia* in plaque but not *Streptococcus mutans* [28]. It may take up to 72 h for these species that produce porphyrins in the biofilm that QLF detects to appear in plaque, yet his model was less than 24 h. QLF can be unreliable for disclosing tooth surfaces covered with plaque [29]. However in the study by Amaechi et al. [26], he did report that the organoselenium sealant (Denteshield™) did have excellent retention at 12 months. Hammad et al. [30] also found plaque could still form around the buccal surfaces of the teeth despite the application of an organoselenium sealant.

The number of *Streptococcus mutans* found attached on the chlorhexidine varnish (cervitec plus) group after aging factor at T4, were 6.941 × 10^3^. The results are in agreement with the current literature on chlorhexidine varnishes [15, 31, 32]. Over prolonged periods during the use of chlorhexidine varnishes (cervitec plus), the chlorhexidine leaches out of the material hence reducing the available concentration in the applied varnish over time. The slow chlorhexidine release can continue over a period of 3 months [33] while we aimed for 5 months. Chlorhexidine varnishes are effective over a 3–4 week period [31]. The optimal CHX varnish concentration is 40% [31] while cervitec plus is 1% CHX which rises to 10%CHX on drying on the tooth surface. Cervitec plus also consists of thymol, an essential oil that has been reported to potentiate the action of chlorhexidine, is an antimicrobial and allows for slower release of chlorhexidine over extended periods of time over 3 months [31]. There are contradicting reports about the retention of this varnish on the tooth surface in literature. However these results are not in agreement with Fiorolli [34] who reports that biofilms can’t form in presence of chlorhexidine. This could be because the systematic review focused on chlorhexidine gels and particularly chlorhexidine gluconate or chloride formulations yet in the study we used chlorhexidine diacetate in varnish form.

Further, the control group at T4 had the lowest bacterial mean count of 7.29 × 10^2^. This can be explained by differences in the surface roughness of the teeth in the three groups. Aging results in increased surface roughness of dental sealants [35]. Increased surface roughness correlates with increased *Streptococcus mutans* adhesion on to surfaces [36].

### Limitations

1. We were unable to observe the biofilm formed on the teeth using an electron microscope.

## Conclusion

The results of the current study showed that neither application of Organoselenium sealant nor Chlorhexidine diacetate varnish completely eliminated Streptococcus biofilm formation on the tooth surfaces. It also follows that both materials were not effective a day after application and after 5 months of aging factor. Further studies should be conducted to find more effective anti-microbial materials for inhibition of *S. mutans* biofilm.

## Data Availability

The datasets used during the current study are available from the corresponding author on reasonable request. All data analyzed during this study are included in this published article in the form of tables and figures.

## Abbreviations

WSLs: White spot lesions
PCR: Polymerase chain reaction
MSB: Mitis salivaris bacitracin
DNA: Deoxy ribonucleic acid
QLF: Quanntitaive light floroscopy
CHX: Chlorohexidine
*S. mutans*: *Streptoccoccus mutans*
rpm: Rotations per minute
